# Fermentation of Milk into Yoghurt and Cheese Leads to Contrasting Lipid and Glyceride Profiles

**DOI:** 10.3390/nu11092178

**Published:** 2019-09-11

**Authors:** Samuel Furse, Alexandre G. Torres, Albert Koulman

**Affiliations:** 1 Core Metabolomics and Lipidomics Laboratory, Wellcome Trust-MRL Institute of Metabolic Science, University of Cambridge, Level 4 Addenbrooke’s Treatment Centre, Keith Day Road, Cambridge CB2 0QQ, UK; 2 Laboratório de Bioquímica Nutricional e de Alimentos e Laboratório de Química e Bioquímica de Lipídios, Instituto de Química, Universidade Federal do Rio de Janeiro, Cidade Universitária, CT/ Bl. A, Rio de Janeiro 21949-909, Brazil; torres@iq.ufrj.br

**Keywords:** lipidomics, fermented dairy, T2DM, lipid metabolism

## Abstract

There is mounting evidence that the consumption of fermented dairy products such as cheese and yoghurt is associated with a reduced risk of type II diabetes. This effect is greater than in fresh milk and differs between cheese and yoghurt. However, the molecular components responsible for the effect are not known. We tested the hypothesis that the lipid and/or glyceride profiles of yoghurts and cheeses are distinct from one another and fresh milk. We developed a novel sample preparation technique for high-fat samples that can be used with Direct Infusion–Mass Spectrometry. We found that the lipid and glyceride profiles of cheddars from the UK, Ireland and France, and hard cheeses from Sweden and Italy were similar to one another but distinct from unfermented dairy products. The lipid and glyceride profile of yoghurts was varied and included types that may be similar to fresh milk. Several odd-chain-containing triglycerides were more abundant, while a variety of others were less abundant, in fermented milk samples. Phosphatidylcholines and phosphatidylethanolamines were more abundant in cheeses, with evidence that the phosphatidylethanomine profile is re-modelled in a way that reflects the bacterial cell envelope. We concluded that a combination of microorganismal metabolism, concentration of the lipid/glyceride fraction and oxidation during fermentation contribute to the observed lipid profile if fermented dairy foods. These differences in the lipid and glyceride profile provide a new avenue for understanding why different fermented dairy foods show a different association with reduced disease risk compared to unfermented dairy.

## 1. Introduction

Epidemiological studies, using dietary assessment approaches have shown that a high intake of fermented dairy foods, particularly from yoghurt and cheese [1,2,3], is associated with a lower risk of type II Diabetes Mellitus (T2DM) [4,5,6,7,8].

These observations have led to investigations of the molecular mechanisms that may drive this association. Evidence from feeding studies has shown that consumption of milk [9], live yoghurt [10,11,12,13,14], cheese [15] and indeed intake of dairy-derived foods generally [16,17] is linked to shifts in the lipoprotein profile in plasma. However, at least one larger study has found that the positive effects are not universal amongst dairy foods, leading them to suggest that participants should be stratified by the type of dairy product they consume [17]. There are no studies published with sufficient power that were able to assess the effect of a particular fermented dairy food.

It is to be expected that fresh and fermented dairy products vary in the amount and type of both fats and lipids that they contain. Butter comprises 80% triglycerides where skimmed milk less than 1%. One may expect this diversity to be at least as wide in the fermentation to produce different cheeses as they are typically fermented by entirely different micro-organismal cultures and for a longer time than yoghurt. This raises questions firstly about which molecular components are associated with a lower risk of T2DM and secondly, how the dairy products they come from may differ.

Tantalisingly, the beneficial effects of the consumption of (fermented) milk products has been linked directly with a small number of fatty acids [18,19]. Specifically, FA(15:0) [20] and conjugated linoleic acid (CLA) [21,22,23,24,25]. As both FA(15:0) and CLA are typically associated with bacterial [26,27,28] rather than mammalian metabolism, we formulated the hypothesis that the lipid and/or glyceride profiles of yoghurts and matured cheeses are distinct from one another and readily identifiable using molecular profiling. Testing this hypothesis is important because it not only provides evidence for wider questions (a possible mechanism in the association between ingestion of dairy fat and lower risk of T2DM), but it may also inform research of human metabolism beyond T2DM. For example, there is a growing body of evidence showing that the profile of triglycerides in the maternal circulation has a bearing on the composition of human milk [29,30] and that how infants are fed can influence how their lipid metabolism develops [29,31].

We tested the primary hypothesis of the study by comparing the molecular profiles of a variety of mild and matured cheeses (five EU countries) and several types of yoghurt (Greek, low fat Greek, organic live, high protein, plain natural) to one another, and to fresh milk. Direct infusion mass spectrometry [32,33] (DI-MS) was used to profile their lipid fractions and fatty acid profile in molecular detail. Novel methods were developed for preparing high-fat samples so that their lipid, glyceride and FA profiles could be acquired using a single method.

## 2. Materials and Methods

*Reagents*—Solvents were purchased from *Sigma-Aldrich Ltd.* (Dorset, UK) of at least HPLC grade and were not purified further. Lipid standards were purchased from *Avanti Polar lipids* (Alabaster, AL; via Instruchemie, Delfzijl, The Netherlands) and used without purification. Consumables were purchased from Sarstedt AG & Co (Leicester, UK) or Wolf Labs (Wolverhampton, UK).

*Sample acquisition and preparation*—Mild and mature cheddar cheeses (30%–40% fat; from the UK, Ireland, France), Parmigiano Reggiano (28.5% fat; Italy) and Vasterbottensost (35% fat; Sweden) and low and high fat yoghurts (0.1%–10% fat; from the UK) were purchased from British Supermarkets (Tesco PLC, Welwyn Garden City, UK; J Sainsbury PLC, London, UK; Ocado Retail Ltd., Hatfield, UK) in November 2018 and February 2019. Samples were aliquoted and stored at −20 °C. Unhomogenised double cream (40% fat) was churned at room temperature using a spatula until separation of the fat and aqueous phases (10 min). The resulting fluids presented as butter and buttermilk and were analysed alongside cream in the present work to illustrate the effects of a physical process in contrast to the fermented dairy samples. Cheese and fatty yoghurt samples (100 mg) were dispersed in a stock solution of guanidinium chloride (6 M) and thiourea (1.5 M) (500 µL, known as GCTU buffer) and freeze-thawed once before extraction of the lipid fraction. Milk and fat-free yoghurt were not treated.

*Extraction of the lipid fraction*—A method for extracting the lipid and triglyceride fraction in a high throughput manner, described recently, was used in the present study [33]. The procedures facilitated the profiling of both the phospholipid (PL) and glyceride (triglyceride and diglyceride, TG/DG) fractions in a high throughput manner. This is challenging in dairy samples as triglycerides typically dominate (>98%) and have distinct physico-chemical properties to the minor phospholipid component. Briefly, the solution of milk/fat-free yoghurt or dispersion of cheese in GCTU buffer (40 µL, prepared as above) was pipetted into a well (96w plate, Esslab Plate+™, 2·4 mL/well, glass-coated) followed by internal standards (150 µL, Mixture of Internal Standards in methanol (See Table S1), water (500 µL) and DMT (500 µL, dichloromethane, methanol and triethylammonium chloride, 3:1:0.002). The mixture was agitated (96 channel pipette) before being centrifuged (3200× *g*, 2 min). A portion of the organic solution (20 µL) was transferred to a high throughput plate (384 w, glass-coated, Esslab Plate+™) before being dried (N_2 (g)_). The samples were transferred immediately to the high throughput plate and dried (N_2 (g)_). The dried films were re-dissolved (TBME, 30 µL/well) and diluted with a stock mixture of alcohols and ammonium acetate (100 µL/well; propan-2-ol: Methanol, 2:1; CH_3_COO.NH_4_ 7.5 mM). The analytical plate was heat-sealed and run immediately.

*Profiling of lipid isolates and data acquisition*—Direct Infusion Mass Spectrometry was used to profile the glycerides, phospholipids and fatty acids separately, using a three-part method described previously [33]. The same study found that the glyceride fraction in some fresh milks and in infant formula typically represents 99.9% of signal intensity in positive ionisation mode and around 35% of that in negative ionisation mode, and thus suppresses the signals of PL variables. This led us to remove glycerides using hexane in studies of fresh milk and infant formula [33], however as samples in the present study did not typically exceed 99.5% TGs washing with hexane did not greatly improve resolution in the present samples. Thus, the samples not washed with hexane were profiled.

All samples were infused into an Exactive Orbitrap (Thermo, Hemel Hampstead, UK), using a Triversa Nanomate (Advion, Ithaca, NY, USA). The Nanomate infusion mandrel was used to pierce the seal of each well before an aliquot of the solution (15 μL) was collected with an air gap (1·5 μL). The tip was pressed against a fresh nozzle and the sample was dispensed using 0·2 psi (N_2 (g)_). Ionisation was achieved at a 1·2 kV. The Exactive started acquiring data 20 s after sample aspiration began. The Exactive acquired data with a scan rate of 1 Hz (resulting in a mass resolution of 65,000 full width at half-maximum (fwhm) at 400 *m*/*z*). After 72 s of acquisition in positive mode the Nanomate and the Exactive switched over to negative mode, decreasing the voltage to −1·5 kV. The spray was maintained for another 66 s, after which Collision-Induced Dissociation commenced, with a mass window of 50–1000 Da, and was stopped after another 66 s. The analysis was then stopped, and the tip discarded before the analysis of the next sample began. The sample plate was kept at 10 °C throughout the data acquisition. Samples were run in row order and repeated several times, if necessary, to ensure accuracy.

*Data collection and handling*—The DI-MS method used in this study was based on an existing method [32] that measured in both positive and negative ion modes with an added third section, in which collision-induced dissociation (CID) was used in a second negative mode [33]. This additional section focuses on the fatty acid profile of the phospholipid fraction as triglycerides do not ionise well in negative mode. Two types of quality control (QC) samples were used. One set consisted of English cheddar dispersed in GCTU solution (*vide supra*). The second has been described [33] and consisted of three infant formulae (soya-, caprine-, bovine-based, 1:1:1) dispersed in Jersey milk (150 mg/mL), that was freeze-thawed once before use.

Positive mode processing used a deviations threshold of 9 ppm. All signals more than twice as strong as the noise (average signal strength across all the blanks for that signal) were carried through. Abundance/Signal intensity in QC samples (0.25, 0.5, 1.0) was correlated against 25%, 50%, 100%, with remaining variables passing if they correlated >0.75. Variables with 0% values across all samples were removed, and finally, the intensities for each sample were normalised to a total of 1000. Negative mode processing used a deviations threshold of 9 ppm and a signal strength threshold of 2. Abundance/Signal intensity in QC samples (0.25, 0.5, 1.0) was correlated against 25%, 50%, 100%, with variables passing if they correlated >0.75. Variables with 0% values across all samples were removed, and finally, the intensities for each sample were normalised to a total of 1000. Processing of the negative mode with CID used a deviations threshold of 9 ppm on a list of fatty acids of chain length 14 to 36 with up to six olefin bonds or one hydroxyl group or both. All signals stronger than noise were carried forward, except those that were fewer than 15% present (i.e., <85% 0 s). This resulted in the discovery of a maximum of 407 variables in positive mode, 1232 in negative mode and 63 fatty acids/hydroxylated FA derivatives.

*Statistical Tests*—Univariate statistical tests were carried out using Microsoft Excel 2013 and Principal Component Analyses (PCAs) carried out using Metaboanalyst 4.0 [34]. PCAs were used to identify which samples grouped together in a data-driven manner. Significance (FDR correction) was assessed using a Bonferroni-corrected *p*-value threshold, based on the number of independent variables for each comparison (1519 for cheeses, 1574 for yoghurt).

## 3. Results

**Figure 1:**
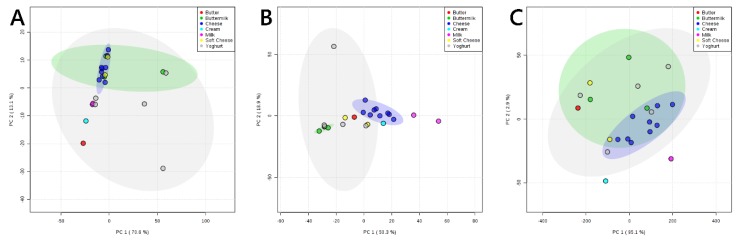
PCAs of samples of fresh and fermented dairy samples used in the present study. Panel (**A**), PCA of signals of dairy samples collected in positive ion mode; (**B**), PCA of signals of dairy samples collected in negative ion mode; (**C**), PCA of signals of fatty acids from dairy samples collected in negative ion mode with collision/induced dissociation. 95% probability boundaries for yoghurt (grey), buttermilk (green) and cheese (dark blue) are shown.

We began by investigating the variety and groupings of the lipid and glyceride profiles of both fresh and fermented dairy products (Figure 1). This indicated to us that the glyceride (Figure 1A), lipid (Figure 1B) and fatty acid (Figure 1C) profiles of cheeses were generally quite similar to one another but distinct from both milk and butter. Soft cheese appears to be relatively similar to matured cheeses. Buttermilk (British, Dutch and Polish) and yoghurt (Greek, low fat, high protein, natural) are the most varied; however, the contiguous distribution of buttermilk samples in negative mode (Figure 1B) suggests that despite the different sources of these products, the phospholipid profile is relatively similar. This is in contrast to the glyceride (Figure 1A) and fatty acid (Figure 1C) profiles of the same. Principal component 2 appears to find a clear distinction between the glyceride profile of cheese and cream/butter (Figure 1A).

**Figure 2:**
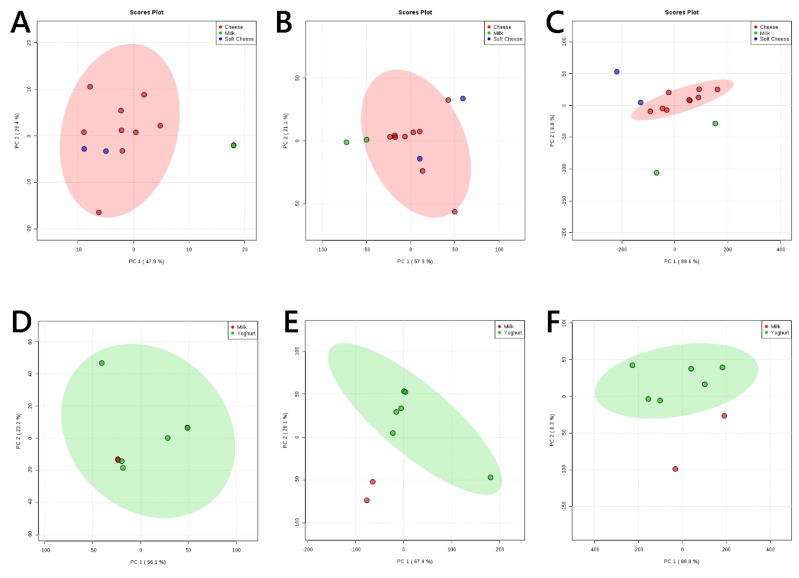
Principal component analyses of sampls of milk with soft and matured cheeses, and yoghurt. Panel (**A**), positive ionisation mode (99% glycerides); (**B**), negative ion mode (>85% phospholipids); (**C**), negative ionisation mode (fatty acids only). Principal component analyses of samples of milk with yoghurt. Panel (**D**), positive ionisation mode (99% glycerides); (**E**), negative ion mode (>85% phospholipids); (**F**), negative ionisation mode (fatty acids only). 95% probability boundaries for cheese (red, panels **A**–**C**) and yoghurt (green, panels **D**–**F**) shown.

These results led us to investigate the difference between milk (the starting material for all fermented dairy products) and yoghurt, soft cheese and matured cheese. A close inspection of the difference between milk and the cheeses showed that the glyceride profile of soft cheese is much more similar to mature cheese than it is to milk (Figure 2A). The phospholipid (Figure 2B) and phospholipid fatty acid (Figure 2C) profiles of mature cheeses are remarkably similar to one another and both are distinct from milk. The fatty acid profile of phospholipids in soft cheeses may also have a marked difference to both milk and matured cheese. The glyceride profiles of yoghurt are varied but not generally distinguishable from milk (Figure 2D), in contrast to the phospholipid (Figure 2E) and phospholipid fatty acid (Figure 2F) profiles, which appear to be distinct.

**Figure 3:**
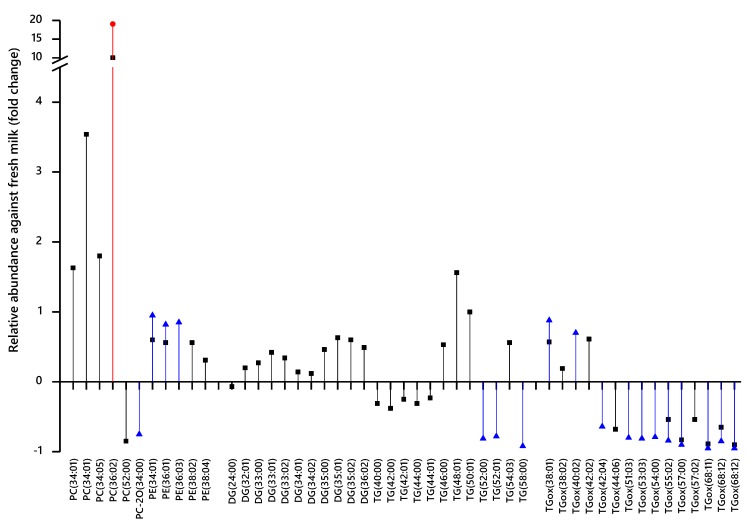
Candidate biomarkers (CBMs) that distinguish milk and either cheddar cheese (black squares), soft cheese (red circle) or yoghurt (blue triangle). Calculations based on 1 subtracted from the mean abundance of the variable for milk divided by that of the mean of the experimental group. Only variables that had both a high (>0.01) or low (<−0.01) loading and passed at the Bonferroni-corrected p-value threshold for significance based on the number of independent variables for each comparison (1519 for cheeses, 1574 for yoghurt) are shown. PC(34:01) and TGox(68:12) were recorded in both positive ionisation and negative ionisation modes. DG CBMs represent species that have lost one equivalent of water, suggesting that they have arisen from fragmentation of TGs during ionisation. DG, diglyceride-H_2_O; PC-2O dialkyl phosphatidylcholine; PC, phosphatidylcholine; PE, phosphatidylethanolamine; TG, triglyceride; TGox, oxidised triglyceride.

These data led us to investigate exactly which variables differed between these samples. We therefore used a supervised multivariate analysis (Partial Least Squares Discriminant Analysis, PLS-DA) to identify which variables distinguish the variables distinguish the fermented milk samples from fresh milk (control). The variables whose abundance was significantly different (student’s T-test), using a corrected threshold for significance, were regarded as candidate biomarkers (Figure 3). This analysis indicated that the profile of glycerides is modulated, with triglycerides possessing the longest fatty acid residues (FARs) being lost from cheeses and yoghurts altogether (Figure 3). Several isoforms of TG comprising FAs with an odd number of carbons are significantly more abundant in fermented milk samples (Figure 3). A variety of oxidised triglycerides (TGox) appear to be much less abundant in fermented milk samples, particularly TGox with the longest polyunsaturated FARs. However, several saturated ones appear to have been oxidised as well (Figure 3). It is important to note that high resolution mass spectrometry enables the separation of lipids to a molecular formula level but not beyond (it cannot separate isobaric species). The resolution of 60,000 at 400 *m*/*z* as used in our set-up allows for the baseline separation of, for instance, molecular formulae C_41_H_76_NPO_8_ + H^+^ (*m*/*z* 742.538) and C_42_H_80_NPO_7_ + H^+^ (*m*/*z* 744.574) but is unable to determine if C_41_H_76_NPO_8_ + H^+^ is PC(33:3) or PE(36:3). Further detailed identifications were not pursued.

Furthermore, we also note that the most abundant isoforms of phosphatidylcholine (PC) and phosphatidylethanolamine (PE) in mammalian samples such as milk are more than 50% more abundant in cheese than in milk (Figure 3). The increase in abundance of PC may be ascribed to a combination of the inability of bacterial and/or fungal cells to absorb and/or metabolise it and the concentration effect of the fermentation. PC(34:01) and PC(36:02) are amongst the most abundant in fresh animal milk, as are isoforms of PE are 34:01, 36:01, and 38:02 [33]. As bacteria do not typically produce PC *de novo*, the increase in the abundance of PCs with respect to milk indicates that fermentation has a concentrating effect on the lipid fraction. This is consistent with the percentage of fat in milk with respect to yoghurts (up to 10%) and mature cheeses (30%–40%).

## 4. Discussion

In this study, the lipid and glyceride profiles of several yoghurts and matured cheeses were acquired and compared with that of fresh jersey milk. This showed that the relative abundance of a number of lipid and glyceride species is shifted during the fermentation process but that this differs between the fermentation to produce yoghurt and the fermentation to produce cheese. There is also evidence for a concentrating effect, with TGs being around twice as abundant in yoghurt as they are in ordinary fresh milk and typically ten times as abundant in matured cheese. This produces a variety of effects.

For example, the lower abundance of TGs with 40–44 FA carbons and 0–2 olefin bonds in cheeses and higher abundance of TGs with 50–58 FA carbons and 0–1 double bonds (Figure 3) indicates that medium chain fatty acids of TGs are consumed during fermentation, despite the concentrating effect of the process.

TGs comprising FARs with an odd number of carbons (typically 15 or 17) were more abundant in cheese samples, as indicated by the presence of diglycerides fragments with odd numbers of FA carbons. This suggests that TGs comprising FAs with an odd number of carbons are either not metabolised by bacteria, are not metabolised as rapidly as even-chain-containing TGs or are also produced by bacteria. Gram-positive bacteria are capable of producing branched FAs with an odd number of carbons [35]; however, they are not able to store TGs, and thus, wild-type strains do not typically produce them. Furthermore, it is not clear why TGs comprising odd-chain FAs might not be used as rapidly as ones containing only even-chains.

The most abundant isoforms of PE in milk are 34:01, 36:01, 36:02 and 38:03, with PE(36:02) being more abundant than all other isoforms of PE put together. However, PE(36:02) does not appear to be significantly more or less abundant in fermented milk products. These configurations were identified using DI-MS, a high-resolution mass spectrometry approach that identifies signals with that may contain several isobaric species. This means that some isoforms of PC that comprise an FA with an odd number of carbons are isobaric with PEs that comprise two FAs that have an overall even number of carbons in their FA residue (and *vice versa*). As the FA profile of phospholipids consists of around 2.5% FAs with odd numbers of carbons, we expect a proportion of all lipids to contain such FAs as a minor component. Furthermore, the isoforms of PE that are more abundant in fermented milk samples (30:00, 34:01, 36:01) are also ones that one may expect to find in either or both Gram-positive and Gram-negative bacteria, in which branched FAs are particularly abundant [35,36]. The FA profile of *Escherichia coli* and *Listeria innocua*, measured from WT strains of these bacteria, grown under laboratory conditionsin the present study, suggest that up to 5% of such FAs may contain an odd number of carbons (Table S2). The abundance of FA(15:0) in fermented milk samples (0.3%–1.0%, Table S3) is no more than that in Gram-positive bacterium *L. innocua* (4%, Table S2). The presence of odd-chain-containing PCs notwithstanding, these observations suggest that the PE fraction is re-modelled to reflect that of the cell envelope of bacteria.

However, there are important differences between the modulation in lipid/glyceride profile associated with fermentations to produce yoghurts and mature cheeses. The concentration of PCs observed so clearly in cheeses does not appear to occur in yoghurt, suggesting that PC is consumed by yoghurt cultures. Furthermore, the abundance of a number of TGox is lower in yoghurt than milk, suggesting that these cultures can metabolise such species and perhaps suppress their formation. The modulation of the glyceride fraction is also remarkably different, with no odd-chain FAR-containing TGs having a higher relative abundance in yoghurt than milk, though several do in cheeses. This may be partly due to the different extent of concentration; whole jersey milk comprises 5% fat where yoghurt comprises no more than 10% fat, where cheese is typically >30%. The fact that some odd-chain-containing TGs are not less abundant in yoghurt than milk indicates that they are not reduced with respect to the overall fat mass of that food.

The shifts in the abundance of lipids and glycerides in fermented milk foods and the evidence for a protective effect of dairy fat against T2DM naturally raise the question of which molecular components, if any, are responsible. Digestion has a profound effect on molecular profiles, typically destroying low abundance species such as those involved in signalling before they reach the (human) circulation. This assumption leads one to identify more abundant species that are gained or concentrated by fermentation. This study suggests that the species that are more abundant in fermented milk foods include bacterial PEs, mammalian PCs and mammalian TGs that comprise FARs with an odd number of carbon atoms. TGs comprising FARs with an even number of carbons are still abundant but relatively less abundant than they are in milk or other fresh dairy foods such as butter.

The higher abundance of odd-chain-containing TGs in fermented dairy foods such as matured cheese and yoghurt is consistent with the association drawn between these species and the protective effect of dairy fat against the hyperglycaemia associated with T2DM, previously [12]. The differing lipid and glyceride profiles between yoghurt and cheeses is consistent with suggestions that participants in metabolic studies who consume such foods should be stratified according to their intake of fermented dairy foods [17].

Some of the species identified as being more abundant in fermented samples may comprise linoleic acid residue(s) (FA(18:2)), such as PC(36:02), PE(38:02), PE(38:04) and TG(51:05). The abundance of FA(18:2) is around 5% in PLs in cheese, and 5%–10% in yoghurts (Table S3). This may be important as evidence for a relationship between ingestion of geometric isomers of conjugated linoleic acid (CLA) and glycaemic control in humans [21,22,23,24,25]. There is also evidence of CLA in both dairy foods [37] and cultures that are used to ferment milk into cheeses and yoghurt [26,27,28]. However, mass spectrometric techniques alone are not able to determine or quantify the types or abundance or geometric isomers of fatty acids, and so further work is required to establish which isomers are present and how much, before conclusions can be drawn about their efficacy.

Further work is required to establish the precise role of species that appear in fermented dairy foods, *in vivo*. For example, odd-chain-containing are also found in human milk from women who eat virtually no dairy foods at all, and very little fresh meat of any kind [29,38]. This suggests that such TGs are a normal part of the biosynthesis of milk glycerides and have a role in infant nutrition. There is already evidence that odd-chain-containing TGs are associated with the abundance of lipids associated with infant growth [29].

## 5. Conclusions

This study shows that the lipid and glyceride profiles of milk fermentation products differ both from milk and one another. A possible role for species comprising odd-chain FAs in protecting humans against T2DM is consistent with the association of dairy foods with a lower risk of T2DM. It is also consistent with the concentration of these species in fermented milk foods. The survival of these species through fermentation is consistent with their particular combination of chemistry and biochemistry; as saturated species, they are not typically sensitive to oxidation and they also appear not to be a prime source of carbon for bacteria. However, it is not yet clear what their activity is *in vivo*.

## Supplementary Materials

The following are available online at https://www.mdpi.com/2072-6643/11/9/2178/s1, Figure S1: Variables that differ significantly in abundance between milk and either cheese, soft cheese or yoghurt, Table S1: Full list of internal standards used, Table S2: The Fatty Acid profile of *E. coli* and *L innocua*, grown under laboratory conditions, Table S3: Full signals file of all species identified in the present study. The full signals data spreadsheets have also been uploaded to MetaboLights, accession number MTBLS1102, www.ebi.ac.uk/metabolights/MTBLS1102.
