# Supplementary material for: Fermentation of Milk into Yoghurt and Cheese Leads to Contrasting Lipid and Glyceride Profiles
Source: Nutrients. 2019 Sep 11;11(9):2178. doi: 10.3390/nu11092178 (PMC6770487; doi:10.3390/nu11092178)
Supplement: Supplementary file 1 [file nutrients-11-02178-s001.zip › Figure S1 -- all variables that pass Ttest.docx]

*Figure S1. Variables that differ significantly in abundance between milk and either cheese, soft cheese or yoghurt. Panel A, Glyceride variables; B, oxidised triglyceride variables; C, phospholipid variables. DG variables represent species that have lost one equivalent of water, suggesting that they have arisen from fragmentation of TGs during ionisation. Calculations based on one subtracted from the mean abundance of the variable for milk, divided by that of the mean of the experimental group (subtracting from 100%). Only variables that pass at the Bonferroni-corrected p-value threshold for significance, based on the number of independent variables for each comparison (1,519 for cheeses, 1,574 for yoghurt) are shown. TGox(52:07) was recorded in both positive and negative ionisation modes. DG, diglyceride-H_2_O; LPE,* lyso*-phoshatidylethanolamine*; *PA-P, phosphatidic acid plasmalogen; PC, phophatidylcholine; PE, phosphatidylethanolamine; SM, sphingomyelin; TG, triglyceride; TGox, oxidised triglyceride.*
