# Supplementary material for: Fermentation of Milk into Yoghurt and Cheese Leads to Contrasting Lipid and Glyceride Profiles
Source: Nutrients. 2019 Sep 11;11(9):2178. doi: 10.3390/nu11092178 (PMC6770487; doi:10.3390/nu11092178)
Supplement: Supplementary file 1 [file nutrients-11-02178-s001.zip › Table S1-- internal standards.docx]

Table S1

| Lipid Class | Isoform |
| --- | --- |
| Cholesteryl ester | CE(18:0-d_6_ ) |
| Ceramide | C16-d_31_ Ceramide |
| Fatty acid | C15:0-d_29_ FA |
| Fatty acid | C17:0-d_33_ FA |
| Fatty acid | C20:0-d_39_ FA |
| lyso-Phosphatidylcholine | lysoPC(C14:0)-d_42_ |
| Phosphatidic acid | PA(C16:0-d_31_/C18:1) Na^+^ salt |
| Phosphatidylcholine | PC(C16:0-d_31_/C18:1) |
| Phosphatidylethanolamine | PE(C16:0-d_31_/C18:1) |
| Phosphatidylglycerol | PG(C16:0-d_31_/C18:1) Na^+^ salt |
| Phosphatidylinositol | PI(C16:0-d_31_/C18:1) NH_4_^+^ salt |
| Phosphatidylserine | PS(C16:0-d_62_) Na^+^ salt |
| Sphingomyelin | SM(C16:0-d_31_) |
| Triglyceride | TG(45:0-d_29_) |
| Triglyceride | TG(48:0-d_31_) |
| Triglyceride | TG(54:0-d_35_) |

Table S1. Table of the Internal standards used in this study.
